# Nurse-Training in Special Hospitals

**Published:** 1920-05-22

**Authors:** Bernard Gilbert

**Affiliations:** Secretary. The Industrial Orthopædic Society, Manor House Hospital, Hampstead, N.W. 3.


*To the Editor of* The Hospital.

### Nurse-Training in Special Hospitals.

*To the Editor of* The Hospital.

Sir,—May I be permitted, through the medium of your paper, to make public a matter which is assuming serious proportions at this hospital, and to ask whether you, or some of your readers, can make any suggestions which will obviate the trouble? This hospital, which is an orthopædic institution for the treatment of pensioners and injured industrial workers, is a purely surgical one, and we find it ever increasingly difficult to obtain nurses, as we cannot offer them a general training. During the war we obtained V.A.D.s, but the supply of these is now inadequate, and there is nobody to take their places.

The only course left seems to be to come to an arrangement with a hospital which specialises in some particular branch—*i.e.*, a fever or medical hospital—by which they send us probationers to undergo training in general surgery. We could offer them lectures in surgical nursing, in addition to the usual training, and they would be thus enabled to obtain a more useful certificate, at the end of their probation, than if they had confined their studies to one branch only. I should very much welcome any alternative suggestion, or any help in bringing this tentative scheme into action, and thank you for your courtesy in permitting me to use your columns.—Yours faithfully,

Bernard Gilbert, Secretary.

The Industrial Orthopædic Society,
Manor House Hospital, Hampstead, N.W. 3.
May 5, 1920.

